# *Notes from the Field:* Misidentification of a Specimen as *Neisseria meningitidis* During a University Classroom Laboratory Exercise — Utah, 2025

**DOI:** 10.15585/mmwr.mm7528a2

**Published:** 2026-07-23

**Authors:** Anna Jones, Clarissa Keisling, Kelly F. Oakeson, Kim Christensen, Liana Au, Michael Stanfill, Leisha D. Nolen

**Affiliations:** ^1^Utah Department of Health and Human Services; ^2^Epidemic Intelligence Service, CDC; ^3^Utah Public Health Laboratory; ^4^Brigham Young University, Provo, Utah.

**Summary**

**What is already known about this topic?**

Any exposure to *Neisseria meningitidis, *including in a laboratory, can result in severe illness and death. Appropriate handling practices and containment conditions are essential to preventing laboratory exposure.

**What is added by this report?**

Deficient microbiology stockroom practices resulted in concern that university microbiology classroom students and employees might have been exposed to *N. meningitidis*, prompting administration of postexposure prophylaxis to 32 persons, two of whom also received lumbar punctures. Subsequent testing identified the organism as *Neisseria sicca, *a nonpathogenic *Neisseria* species.

**What are the implications for public health practice?**

Laboratory safety protocols in university or instructional settings, including inventory controls and appropriate supervision of laboratory staff members, are important for preventing potential exposures and unnecessary medical interventions.

On October 2, 2025, the Utah Department of Health and Human Services (DHHS) received notification of multiple potential exposures to *Neisseria meningitidis* in a university microbiology stockroom and laboratory classroom beginning on September 22. Among the human-restricted *Neisseria* species (*1*), *N. meningitidis* and *Neisseria gonorrhoeae* are potentially pathogenic; other species are typically nonpathogenic (*1*). Vaccines offering protection against meningococcal serogroups A, B, C, W, and Y are available (*2*). Irrespective of vaccination status, CDC recommends prompt antibiotic postexposure prophylaxis (PEP) after exposure to *N. meningitidis* (*3*).

## Investigation and Outcomes

### Potential Exposures

The first potential exposure occurred on September 22 (Figure). On that date, microbiology stockroom employees began preparing bacterial isolates for a multiday laboratory experiment in a university microbiology classroom. The experiment involved students using morphologic characterization and biochemical tests to identify the provided unknown bacterium, which was intended to be *Plesiomonas shigelloides.* Employees handled the isolates on multiple instances during September 22–30. Employees wore gloves and laboratory coats but did not use respiratory protection, eye protection, or a Biological Safety Cabinet. 

**FIGURE:**
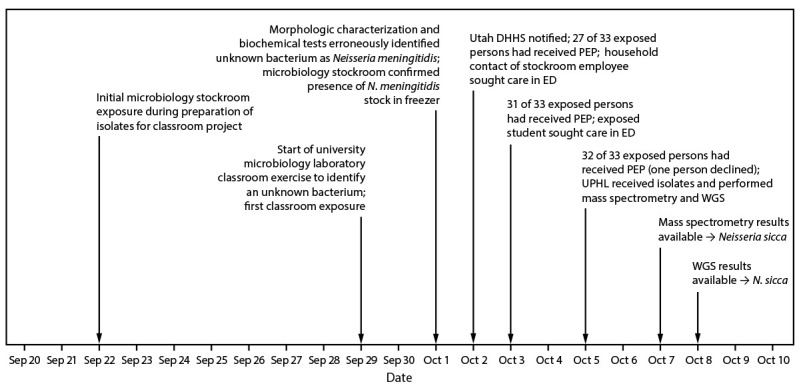
Timeline of potential *Neisseria* *meningitidis* classroom laboratory exposure and response — Utah, September–October 2025 **Abbreviations:** DHHS = Department of Health and Human Services; ED = emergency department; PEP = postexposure prophylaxis; UPHL = Utah Public Health Laboratory; WGS = whole-genome sequencing.

On September 29, laboratory students began working with the isolates in the classroom laboratory wearing gloves and laboratory coats. On October 1, all the students identified the stockroom-provided unknown bacterium as *N. meningitidis*, rather than the expected *P. shigelloides*. The bacterium was a gram-negative, oxidase- and catalase-positive diplococcus with carbohydrate fermentation patterns consistent with *N. meningitidis**.*

### Initiation of Public Health Response

The stockroom freezer was found to contain a vial labeled *N. meningitidis,* although the university did not test the contents before disposing of it. Public health officials quickly evaluated risk among potentially exposed persons, tested samples, provided public health recommendations, and assisted with the investigation. This evaluation was deemed to be an authorized public health response activity by the Utah DHHS. This activity was reviewed by CDC, deemed not research, and conducted consistent with applicable federal law and CDC policy.*

### Identification of Potentially Exposed Persons and Recommendations for Postexposure Prophylaxis

University officials determined that 33 professors, students, and stockroom employees had manipulated the unknown bacterium during isolate preparation or the classroom project. Fifteen (45%) persons had documentation of previous receipt of ≥1 doses of MenACWY or MenB vaccine. Health officials recommended ciprofloxacin or ceftriaxone postexposure prophylaxis (PEP) and monitoring of clinical status of all potentially exposed persons for 10 days (*3*). Thirty-two (97%) persons received PEP within 14 days; one person declined. Two persons with headache (one exposed student and one household contact of an exposed employee) sought emergency care and received medical evaluations including lumbar puncture. Neither received a diagnosis of *N. meningitidis* infection. No other persons experienced symptoms.

### Public Health Laboratory Identification of Bacterium and Retesting of Sample Provided to Students 

On October 6, the Utah Public Health Laboratory (UPHL) received two of the suspected *N. meningitidis* isolates for testing. On October 7 and 8, mass spectrometry and whole-genome sequencing (WGS), respectively, identified the isolates as *Neisseria sicca*, a bacterium that is commonly found in the upper respiratory tract and is nonpathogenic in immunocompetent persons.

UPHL repeated the carbohydrate fermentation tests conducted by the students and obtained results suggesting *N. meningitidis*. However, in combination with mass spectrometry and WGS results, the *N. sicca* strain was determined to be an atypical carbohydrate fermenter, which had resulted in the misidentification of *N. sicca *as* N. meningitidis*. Because *N. sicca* is nonpathogenic, no further intervention was indicated.

### Review of Stockroom Procedures and Inventory Controls

With assistance from Utah DHHS, the university investigated the stockroom and reviewed its procedures. The investigation found inadequate inventory controls for identifying, labeling, storing, and segregating biological materials according to handling practices and containment conditions. Additional practice gaps included limited stockroom employee supervision, inadequate availability and use of personal protective equipment, and insufficient documentation of sample inventory, disposition, and chain of custody.

## Preliminary Conclusions and Actions

*N. meningitidis *infections (including deaths) acquired through laboratory exposure have been reported (*4*). Although no person was exposed to *N. meningitidis *in this event, persons for whom potential exposure was considered did experience unnecessary interventions, including invasive procedures and antibiotic PEP. After-action review underscored the importance of laboratory and stockroom safety procedures, including inventory controls, to ensure that cultures and stocks are accurately labeled and appropriately stored. These measures could help prevent cross-contamination or misuse of stocks and support appropriate occupational health measures (*5*). This event prompted an overhaul of stockroom documentation, supervision, and training protocols, as well as a careful inventory of existing stocks, with recommendations to separate stocks used for research from those used in a classroom. Instructional laboratories and stockrooms should establish clear guidelines outlining appropriate inventory controls and documentation, the use of safety equipment, and required training in the safe handling of microorganisms to prevent exposure.

## Footnotes

* 45 C.F.R. part 46, 21 C.F.R. part 56; 42 U.S.C. Sect. 241(d); 5 U.S.C. Sect. 552a; 44 U.S.C. Sect. 3501 et seq.
